# ECG challenge: inversion without infarction

**DOI:** 10.1093/ehjcr/ytaf438

**Published:** 2025-09-02

**Authors:** Nithin George

**Affiliations:** Department of Cardiology, Lyell McEwin Hospital, SA 5112, Adelaide, Australia

## Clinical vignette

**Figure 1:**
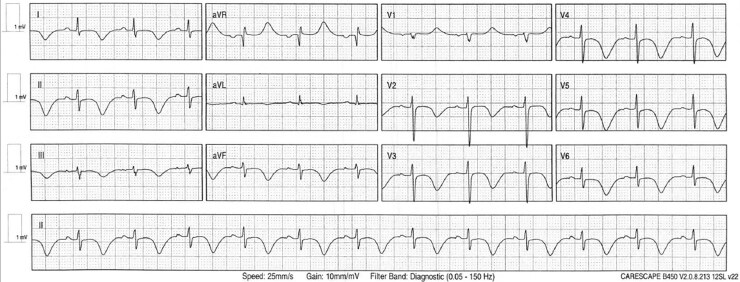
Twelve-lead ECG showing sinus rhythm with deep global T-wave inversions and corrected QTc prolongation (580 ms).

A woman in her mid-40s presented with transient visual disturbances and dizziness following acute emotional stress. Brain imaging revealed an occipital infarct. Cardiac biomarkers were elevated, with high-sensitivity troponin in the 60s (µg/L) and NT-proBNP in the 2700s (ng/L). Transthoracic echocardiography demonstrated apical left ventricular hypokinesis with a small apical thrombus; normal inflammatory markers. The resting 12-lead electrocardiogram (*Figure 1*) on Day 2 showed striking and dynamic repolarization abnormalities.

## Question 1

### What diagnosis is most closely represented in this ECG?

- Apical hypertrophic cardiomyopathy
- Acute anterior ST-elevation myocardial infarction
- Takotsubo cardiomyopathy
- Acute myocarditis
- NSTEMI

Correct answer: C. Takotsubo cardiomyopathy.

### Explanation

Takotsubo cardiomyopathy (TTC) frequently presents with deep, diffuse T-wave inversions and prolonged QTc in the subacute phase, often following an acute emotional or physical stressor.^1^ This dynamic pattern and ECHO evidence of apical hypokinesis strongly favour TTC over apical hypertrophic cardiomyopathy (HCM).^2^ Acute myocardial infarction generally demonstrates territorial ST changes and/or Q-wave evolution, while myocarditis produces non-territorial ST/T abnormalities with inflammatory markers.^3^ The dynamic ECG evolution, ECHO findings, and reversibility of TTC are key clues in distinguishing it from other diagnoses.

## Question 2

### What is the primary mechanism responsible for the prolonged QTc in this patient?

- Myocardial fibrosis
- Myocardial ischaemia due to coronary occlusion
- Intramyocardial oedema and repolarization abnormalities
- Electrolyte imbalance
- Genetic long QT syndrome

Correct answer: C. Intramyocardial oedema and repolarization abnormalities.

### Explanation

In TTC, transient intramyocardial oedema disrupts myocardial ion channel function, delaying repolarization and causing QTc prolongation.^4^ Cardiac MRI (T2-weighted and T1 mapping) typically reveals circumferential myocardial oedema without late gadolinium enhancement, differentiating TTC from structural cardiomyopathies.^5^ Myocardial ischaemia from coronary occlusion would produce territorial ST-segment elevation and infarct-related wall motion abnormalities, while electrolyte disturbances or genetic long QT syndromes usually lack the acute stress trigger and dynamic ECG resolution seen in TTC. QTc prolongation in TTC is often most marked several days after onset and gradually normalizes with the resolution of myocardial oedema.^1^

## Question 3

### What is the most appropriate next management step for this patient’s QTc prolongation?

- Implantation of an Implantable cardioverter-defibrillator (ICD)
- Initiation of amiodarone
- Avoidance of QT-prolonging drugs and serial ECG monitoring
- Emergency coronary angioplasty
- High-dose corticosteroid therapy

Correct answer: C. Avoidance of QT-prolonging drugs and serial ECG monitoring.

### Explanation

In TTC, QTc prolongation is usually transient and reflects reversible repolarization abnormalities rather than a persistent arrhythmic substrate. Management focuses on supportive care: avoiding QT-prolonging drugs, correcting electrolyte imbalances, and monitoring with serial ECGs until QTc normalizes.^1^ ICDs are not indicated unless there is a separate, proven high-risk arrhythmic condition. Amiodarone is avoided because it can further prolong the QT interval. Emergency PCI is relevant only at initial presentation if acute coronary occlusion is suspected; not urgently indicated. High-dose corticosteroids are not part of TTC management unless there is concurrent myocarditis.

**Consent:** Written informed consent was obtained from the patient for publication of clinical information and ECG images. Efforts to protect patient anonymity have been made.

**Funding:** None.

## Data Availability

Data from this case report are available on reasonable request. Please contact the corresponding author.
